# Effects of Diazepam Addition to Standard Treatment of Atrial Fibrillation in Emergency Department Settings: A Unicentric Retrospective Study

**DOI:** 10.3390/medicina62050861

**Published:** 2026-04-30

**Authors:** Kristina Vidović, Josip Krnić, Benjamin Benzon

**Affiliations:** 1 Emergency Department, University Hospital Centre Split, 21000 Split, Croatia; kristina.vidovic@kbsplit.hr (K.V.); josip_krnic@yahoo.com (J.K.); 2 Department of Anesthesiology, Reanimatology and Intensive Care, University Hospital Centre Split, 21000 Split, Croatia; 3 Laboratory for Pathobiology of Tissues, Department of Anatomy, Histology and Embryology, University of Split School of Medicine, 21000 Split, Croatia

**Keywords:** atrial fibrillation, diazepam, benzodiazepines, sympatholysis, rate control, rhythm conversion, emergency department, retrospective cohort study

## Abstract

*Background and Objectives:* Diazepam, a GABA_A_ receptor agonist with sympatholytic properties, is sometimes co-administered with antiarrhythmic agents in the emergency management of atrial fibrillation (AF), yet evidence supporting this practice is remarkably limited. Given the established role of sympathetic activation in the initiation and maintenance of AF, we investigated whether adjunctive diazepam influences treatment outcomes. *Materials and Methods:* This single-centre retrospective cohort study included 72 hemodynamically stable patients presenting with AF to the emergency department of University Hospital Centre Split, Croatia. Patients were stratified by treatment strategy into a rhythm control group (*n* = 33, receiving any Class IC/III antiarrhythmic) and a rate control only group (*n* = 39, beta-blockers and/or digoxin). Diazepam was administered orally at the physician’s discretion (median dose 5 mg). Primary outcomes were rhythm conversion and achievement of a heart rate < 110 bpm. Secondary outcomes included changes in heart rate, blood pressure, and time to therapeutic goal. *Results:* Diazepam was administered to 32 patients (44.4%). In the rate control stratum, spontaneous rhythm conversion was significantly higher with diazepam (40.0% vs. 9.5%; OR 6.33, 95% CI 1.06–37.78, *p* = 0.046), corresponding to a model-predicted increase in conversion probability from 8% to 33%. This effect was absent in the rhythm control group (64.3% vs. 64.7%; OR 0.98, *p* = 1.000). Diazepam increased the odds of achieving HR < 110 bpm by 3.46-fold (95% CrI 0.63–23.1, posterior probability of benefit 92%) in the rate control group. Diazepam-treated patients in the rate control group had longer median time to therapeutic goal (4.2 vs. 2.8 h, *p* = 0.005). In the rhythm control group, diazepam was associated with reduced variability in diastolic blood pressure response (*p* = 0.006). *Conclusions:* Adjunctive diazepam was associated with a significantly higher rate of spontaneous rhythm conversion in AF patients receiving rate control therapy only, consistent with sympatholysis removing a key factor sustaining the arrhythmia. This effect was not observed when Class IC/III antiarrhythmics were co-administered, suggesting that diazepam’s benefit is context-dependent. These hypothesis-generating findings warrant prospective validation, with attention to thromboembolic risk in patients who convert unexpectedly.

## 1. Introduction

Atrial fibrillation (AF) is the most common sustained cardiac arrhythmia and represents a substantial proportion of the cardiac pathology encountered in emergency departments [1]. The pathophysiology of AF involves ectopic triggers, most commonly originating from the pulmonary vein ostia, and multiple re-entrant circuits that are typically a consequence of atrial interstitial fibrosis. The autonomic nervous system plays a central role in both the initiation and maintenance of AF: the sympathetic division, through the actions of adrenaline and noradrenaline, lowers the firing threshold of atrial myocardium, increases automaticity, and raises the likelihood of both early and delayed afterdepolarizations—all of which increase the probability of ectopic triggers that, given a favourable substrate for re-entry, can initiate an AF episode [1]. Beyond initiation, sympathetic innervation contributes to pathological atrial remodelling that renders the myocardium more arrhythmogenic. Furthermore, disordered afferent sympathetic signalling from low-pressure baroreceptors in the fibrillating atrial walls results in increased sympathetic tone, creating a feed-forward loop that promotes both the occurrence and perpetuation of AF episodes [1].

Emergency management of AF relies on two principal strategies: rate control and rhythm control (cardioversion). Rate control is a purely pharmacological approach employing beta-blockers, calcium channel blockers, or cardiac glycosides, while rhythm control may involve pharmacological cardioversion with Class IC or III antiarrhythmics and, in selected cases, electrical cardioversion [1]. The choice of strategy depends on the duration and type of AF, the patient’s hemodynamic status, and the presence of structural heart disease.

Diazepam is a benzodiazepine that acts as a GABA_1_ receptor agonist in the central nervous system, producing anxiolysis, sedation, and muscle relaxation. In the limbic system and thalamus, it initiates a behavioural pattern associated with rest, which is characterised by sympatholysis—a reduction in sympathetic nervous system activity [2,3]. Given the established role of sympathetic activation in the pathogenesis and maintenance of AF, the sympatholytic properties of diazepam provide a rational pharmacological basis for its potential benefit as an adjunctive agent.

In our local clinical practice, diazepam is sometimes co-administered with antiarrhythmic agents in the emergency management of AF, yet the evidence base for this practice is remarkably thin. A literature search reveals only two studies and a small number of case reports addressing the role of diazepam in AF and other arrhythmias. Spracklen et al. [4] administered intravenous diazepam as a hypnotic prior to electrical cardioversion in 111 AF patients, observing no effect of diazepam on AF during the pre-shock observation period; however, the study did not report whether patients had received antiarrhythmic agents beforehand or the duration of observation. Kumagai et al. [5] conducted an electrophysiological study on 18 patients (only two with AF) after a washout period from prior antiarrhythmics, finding that intravenous diazepam at 0.2 mg/kg had a sympathomimetic effect—specifically, increased atrioventricular nodal conduction and shortened sinus cycle length. Van Loon [6] reported isolated cases of ventricular arrhythmias responding to diazepam.

Given the paucity of evidence and the mechanistic rationale linking sympatholysis to AF termination, we undertook a retrospective cohort study to compare treatment outcomes in AF patients who received diazepam as an adjunct to antiarrhythmic therapy with those who did not.

## 2. Materials and Methods

### 2.1. Patients and Setting

This was a single-centre retrospective cohort study conducted in a one-year period, at the Emergency Department of University Hospital Centre Split (KBC Split), Split, Croatia. The study included consecutive patients presenting with atrial fibrillation who were managed according to the department’s AF management protocol. Inclusion criteria were age ≥ 18 years and hemodynamic stability (i.e., absence of the need for immediate electrical cardioversion).

The institutional AF management protocol governed the treatment of all included patients. On presentation, AF was classified as newly diagnosed, paroxysmal (self-terminating within 7 days, with most episodes lasting < 48 h), persistent (duration > 7 days), or permanent. Hemodynamically unstable patients were referred for immediate electrical cardioversion (ECV). Hemodynamically stable patients were managed with either a rate control approach or a rhythm control approach (if AF onset was within 24 h).

For rate control, the target was a resting heart rate below 110 beats per minute (bpm), with stricter targets in symptomatic patients; the mainstay agent in our setting was bisoprolol, alone or in combination with digoxin. For rhythm control, the choice of agent depended on the presence and type of structural heart disease. Amiodarone was indicated for patients with heart failure with reduced ejection fraction (LVEF ≤ 40%), stable heart failure with mildly reduced ejection fraction (LVEF 41–49%), coronary artery disease, or valvular heart disease. Flecainide or propafenone (intravenous or oral) was the agent of choice for patients with structurally normal hearts, with concomitant beta-blocker, diltiazem, or verapamil therapy recommended to prevent 1:1 atrial flutter conduction. If AF duration exceeded 24 h or was of unknown duration, transoesophageal echocardiography was required before cardioversion. All patients undergoing cardioversion received anticoagulant therapy for at least the next 4 weeks, regardless of CHA_2_DS_2_-VASc score.

Discharge criteria were reaching rate or rhythm control, or, if those were not reached, then after 24 h of observation at the ER, where patients were transferred.

Diazepam was administered orally at the treating physician’s discretion as an adjunctive agent with the antiarrhythmic regimen. The decision to administer diazepam was not governed by formal protocol criteria and reflected clinical practice at the institution.

### 2.2. Data Collection

Patient data were retrospectively extracted from emergency department records. The following variables were collected: demographic characteristics (age, sex, and self-reported height and weight), baseline vital signs (heart rate, systolic and diastolic blood pressure), type and dose of antiarrhythmic agents administered, diazepam use and dose, rhythm on final assessment, along with blood pressure, and time to achievement of the therapeutic goal. Repeated visits were considered as independent events; missing data points were excluded from analysis.

### 2.3. Outcomes

Primary outcomes (therapeutic goals) were as follows: probability of rhythm conversion (return to sinus rhythm), probability of heart rate < 110 bpm, and probability of heart rate < 100 bpm.

Secondary outcomes included: absolute and relative change in heart rate (ΔHR), absolute and relative change in systolic and diastolic blood pressure (ΔSBP, ΔDBP), and time from treatment initiation to achievement of a therapeutic goal. Patients hospitalised before reaching the therapeutic goal were censored from time-to-goal analyses.

### 2.4. Statistical Analysis

Continuous variables were summarised as median with interquartile range (IQR) and range; categorical variables were summarised as counts with percentages. Binary outcomes were compared using Fisher’s exact test, with odds ratios (ORs) and 95% confidence intervals (CIs) calculated using the log-transform method. Continuous outcomes were compared using the Mann–Whitney U test or Mood’s median test. The F-test for equality of variances was used to compare the variability of hemodynamic responses between groups.

To examine the independent effect of diazepam while accounting for confounders and effect modification, Bayesian logistic regression models were fitted. Prior specifications followed an empirical approach based on the magnitude of effects and uncertainties reported in the literature (see Supplementary Materials on Prior Selection). Results are reported as posterior medians with 95% credible intervals (CrIs) and posterior probabilities of benefit. Internal model validation included goodness-of-fit measures, partial residual plots, and calibration curves. Furthermore, a propensity score matching with inverse probability of treatment weighting (IPWT) was done to validate the main positive findings.

As measures of evidence, R^2^, Bier index, *p*-values, and Bayesian posterior probabilities were used. *p*-values were interpreted in accordance with the recommendations of the ASA Statement on *p*-values [7].

All univariate analyses and raincloud plots were performed in Python 3.12 using Pandas 3.0.2, NumPy 2.4.4., SciPy 1.17.1., Statsmodels 0.14.6 and Matplotlib (standard). Bayesian multivariate logistic regression models were fitted in R (v. 4.4) using the brms (2.22.0) package with Stan 2.32.2 as the backend sampler and visualised in ggplot2 4.0.2. Code was generated by Claude Opus 4.6 (Anthropic, San Francisco, CA, USA), a large language model, and its output was assessed by one of the co-authors (B.B.).

## 3. Results

### 3.1. Patient Characteristics and Treatment Patterns

A total of 72 patients with atrial fibrillation were included in the analysis. The median age was 69 years (IQR 61–75, range 34–92), with equal sex distribution (36 males, 50.0%). The median body mass index was 27.3 kg/m^2^ (IQR 25.9–29.3). At presentation, the median heart rate was 126 bpm (IQR 118–140), the median systolic blood pressure was 140 mmHg (IQR 120–150), and the median diastolic blood pressure was 80 mmHg (IQR 70–90). Baseline characteristics are presented in Table 1.

Diazepam was administered to 32 patients (44.4%), with a median dose of 5 mg (range 2–10 mg). The most commonly used antiarrhythmic agent was bisoprolol (84.7%), followed by digoxin (27.8%), flecainide (22.2%), amiodarone (18.1%), propafenone (5.6%), and carvedilol (1.4%). The majority of patients received two antiarrhythmic agents (54.2%), while 43.1% received monotherapy and 2.8% received three agents (Table S1).

Patients were stratified according to treatment strategy: those receiving any Class IC/III antiarrhythmic were grouped into a rhythm control stratum (*n* = 33, 45.8%), while those receiving beta-blockers with or without digoxin were assigned to a rate control stratum (*n* = 39, 54.2%).

### 3.2. Effect of Diazepam on Heart Rhythm

Among patients who received Class IC or III antiarrhythmics, 16 (≈49%) received diazepam (dzp+) and 17 (≈51%) did not (dzp−). The probability of rhythm conversion was similar in both groups (64.3% in dzp+ vs. 64.7% in dzp−, OR 0.98, 95% CI 0.22–4.30, *p* = 1.000). In contrast, among patients in the rate control stratum, where spontaneous conversion might occur, the difference was substantially larger: 40.0% (6/15) in the diazepam-treated group converted, compared with only 9.5% (2/21) of patients not treated with diazepam (OR 6.33, 95% CI 1.06–37.78, *p* = 0.046). Baseline characteristics and univariate outcomes for both strata are presented in Table 2.

To further examine the effect of diazepam on rhythm conversion while accounting for potential confounders and effect modification, a Bayesian logistic regression model was fitted with treatment strategy (Class IC/III use), diazepam administration, their interaction, age, and sex as predictors (Table S2, Figure S1). After adjustment, Class IC/III antiarrhythmic use was strongly associated with rhythm conversion (OR 6.71, 95% CrI 1.11–47.60, posterior probability of benefit 98%). Diazepam showed a beneficial effect among patients receiving rate control only (OR 3.86, 95% CrI 0.61–23.3, posterior probability of benefit 92%). The model-derived predicted probabilities of rhythm conversion were as follows: rate control alone 7% (95% CrI 2–20%), rate control plus diazepam 22% (95% CrI 6–52%), rhythm control alone 55% (95% CrI 34–74%), and rhythm control plus diazepam 54% (95% CrI 33–74%). Diazepam was thus associated with approximately a 3-fold increase in conversion probability among patients receiving rate control only.

Next, to validate this even further, we did the propensity score (PS) analysis on the rate control stratum. A PS model was constructed with the following variables: age, sex, weight, baseline HR, baseline SBP and digoxin. The PS model achieved a good balance between groups in this stratum (Figure S2). After balancing the estimated effect of diazepam, the results showed OR = 10.36 (95% CI 1.33–80.93, *p* = 0.026).

### 3.3. Effect of Diazepam on Heart Rate

**Figure 1:**
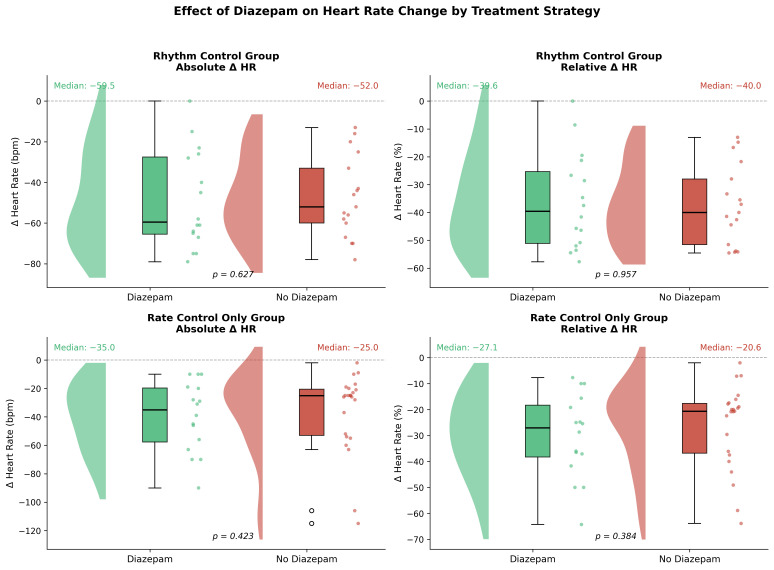
Change in heart rate (ΔHR) by treatment strategy and diazepam use. Raincloud plots show density estimates, box plots (median, IQR, 1.5 × IQR whiskers), and individual observations for the rhythm control (**upper**) and rate control only (**lower**) groups, with absolute (**left**) and relative (**right**) ΔHR. Dashed line: no change from baseline. *p*-values from Mann–Whitney U test. Green: diazepam; red: no diazepam. Abbreviations: ΔHR, change in heart rate; bpm, beats per minute.

In the rhythm control stratum, median absolute heart rate reduction was −59.5 bpm (IQR −65.5 to −27.5) with diazepam versus −52.0 bpm (IQR −60.0 to −33.0) without diazepam (*p* = 0.627). In the rate control stratum, the corresponding values were −35.0 bpm (IQR −57.8 to −19.8) versus −25.0 bpm (IQR −53.0 to −20.5, *p* = 0.423). Relative changes followed a similar pattern (Figure 1).

Regarding therapeutic goals specific to the rate control approach, an HR < 110 bpm was achieved in 93.8% (15/16) of patients treated with diazepam and 78.3% (18/23) of patients not receiving diazepam (OR 4.17, 95% CI 0.44–39.68, *p* = 0.370). Similarly, an HR < 100 bpm was achieved in 87.5% (14/16) of patients in the diazepam group and 65.2% (15/23) of patients not treated with diazepam (OR 3.73, 95% CI 0.67–20.69, *p* = 0.152).

In a multivariate model restricted to the rate control stratum and including age and sex, diazepam increased the odds of achieving HR < 110 bpm by 3.46-fold (95% CrI 0.63–23.1, posterior probability of benefit 92%) (Table S3 and Figure S3).

### 3.4. Effect of Diazepam on Time to Therapeutic Goal

**Figure 2:**
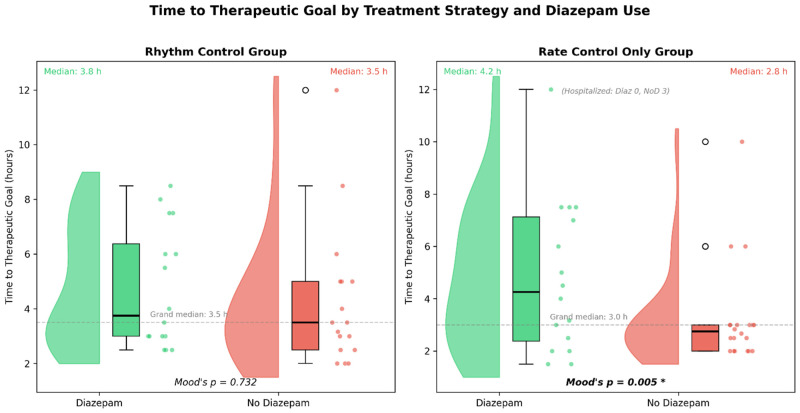
Time to therapeutic goal (heart rate < 110 bpm or rhythm conversion) by treatment strategy and diazepam use. Raincloud plots for the rhythm control (**left**) and rate control only (**right**) groups. Dashed line: grand median. Three no-diazepam patients in the rate control group were hospitalised before reaching the goal (censored). *p*-values: Mood’s median test (rate control only). Green: diazepam; red: no diazepam.

In the rhythm control stratum, time to therapeutic goal was similar between groups (3.8 h [IQR 3.0–6.4] in dzp+ vs. 3.5 h [IQR 2.5–5.0] in dzp−, *p* = 0.415). In the rate control stratum, patients who received diazepam took 4.2 h (IQR 2.4–7.1) to reach HR < 110 bpm or to spontaneously convert, compared with 2.8 h (IQR 2.0–3.0) in those who did not receive diazepam (Mood’s median test *p* = 0.005, Figure 2). Three patients in the no-diazepam rate control group did not reach the therapeutic goal and were admitted to the ICU after 24 h.

### 3.5. Effect of Diazepam on Blood Pressure

**Figure 3:**
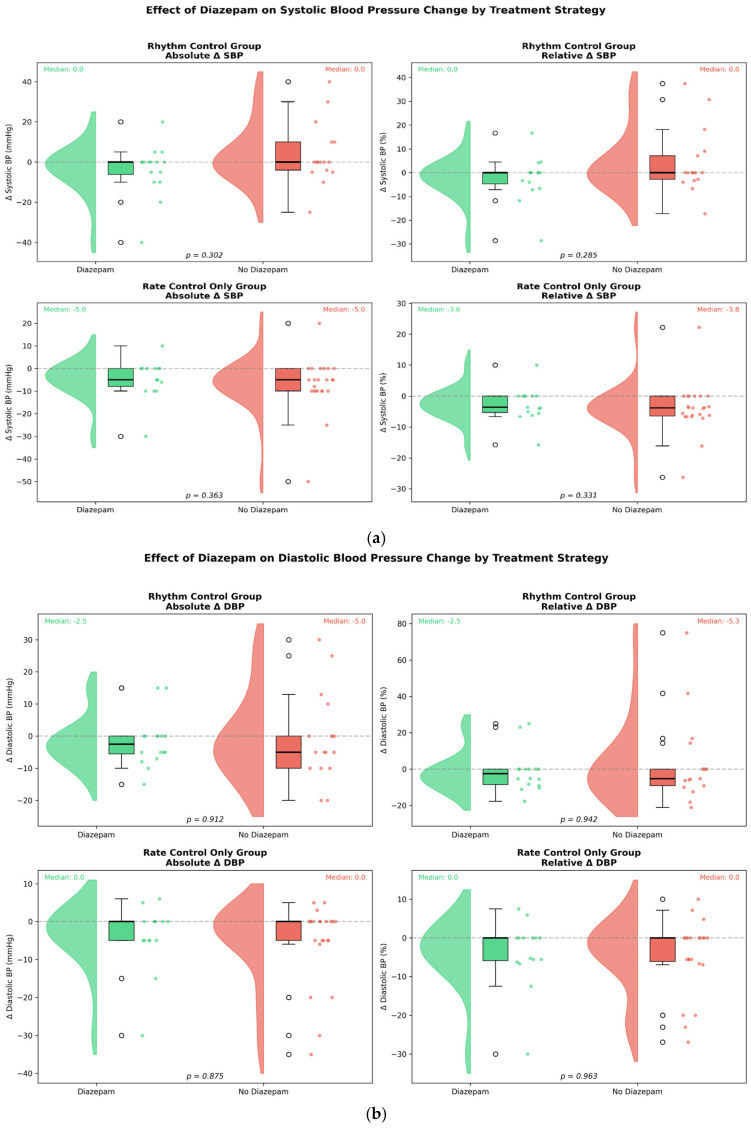
Changes in blood pressure from baseline by treatment strategy and diazepam use. (**a**) Systolic blood pressure (ΔSBP). (**b**) Diastolic blood pressure (ΔDBP). Raincloud plots for rhythm control (**upper**) and rate control only (**lower**) groups, with absolute (**left**) and relative (**right**) changes. In the rhythm control group, ΔDBP variability was significantly lower with diazepam (F-test: *p* = 0.039 absolute; *p* = 0.006 relative). *p*-values from Mann–Whitney U test. Green: diazepam; red: no diazepam. Abbreviations: ΔSBP/ΔDBP, change in systolic/diastolic blood pressure; mmHg, millimetres of mercury.

Blood pressures were analysed as absolute and relative differences between values at admission and values at final assessment before discharge from the emergency department. In the rhythm control stratum, median changes in systolic blood pressure were 0 mmHg in both groups (*p* = 0.302). In the rate control stratum, median systolic blood pressure decreased by 5 mmHg in both groups (*p* = 0.363) (Figure 3a).

Median diastolic blood pressure changes were similar in both groups across both strata (Figure 3b). However, in the rhythm control stratum, both relative and absolute diastolic blood pressure differences showed significantly greater heterogeneity in the no-diazepam group, with a bias toward an increase in diastolic blood pressure (absolute ΔDBP: SD 7.9 vs. 13.7 mmHg, F = 3.00, *p* = 0.039; relative ΔDBP: SD 11.2% vs. 23.6%, F = 4.41, *p* = 0.006).

## 4. Discussion

The principal finding of this study is that adjunctive diazepam administration was associated with a higher rate of spontaneous rhythm conversion among patients with atrial fibrillation receiving rate control agents only. The sympatholytic properties of diazepam offer a plausible mechanistic explanation for these findings. As a GABA_1_ receptor agonist, diazepam reduces sympathetic nervous system activity through its effects on the limbic system and thalamus [2,3]. Since sympathetic activation plays a well-established role in both triggering and sustaining AF—through lowered atrial firing thresholds, increased automaticity, promotion of afterdepolarisations, and pathological atrial remodelling [1]—sympatholysis through diazepam could plausibly remove a key sustaining factor for the arrhythmia.

In patients receiving Class IC or III antiarrhythmics, the pharmacological mechanism of cardioversion—sodium channel blockade (flecainide, propafenone) or potassium channel blockade with action potential prolongation (amiodarone)—directly addresses the electrophysiological substrate of AF. In this context, the additional sympatholytic effect of diazepam is likely redundant; the dominant pharmacological force driving conversion is the antiarrhythmic agent itself.

The potential for diazepam to increase spontaneous rhythm conversion in patients on rate control therapy has several noteworthy clinical implications. Any strategy that increases spontaneous conversion rates requires careful consideration of thromboembolic risk. According to current guidelines, if AF has been present for more than 24–48 h, cardioversion carries a risk of thromboembolism due to potential thrombus formation in the fibrillating atria [1]. Patients who have not been adequately anticoagulated may be at risk if conversion occurs unexpectedly. Furthermore, in patients for whom Class IC/III antiarrhythmic agents are contraindicated and who have a low probability of spontaneous conversion [8]—for example, patients with thyroid disease in whom amiodarone is undesirable due to its substantial iodine content and 14–18% incidence of thyroid dysfunction—a combination of beta-blockers and/or digoxin with diazepam could offer an alternative pathway to rhythm conversion without the risks associated with antiarrhythmic drugs.

An apparently paradoxical finding is that diazepam-treated patients in the rate control stratum had a longer median time to therapeutic goal. This may be due to the fact that 40% of diazepam-treated patients achieved their therapeutic goal through spontaneous rhythm conversion rather than through rapid rate reduction alone. Moreover, the finding by Kumagai et al. [5] that diazepam increases AV node conductivity and shortens sinus cycle length may also contribute to this observation.

When it comes to blood pressure, diazepam appears to have no effect on average change; however, it was associated with a reduction in the proportion of patients whose diastolic pressure rose following treatment in the rhythm control group, suggesting a stabilising effect on hemodynamic variability.

This study has several important limitations that should be considered when interpreting the findings. The retrospective and unicentric design introduces potential selection bias. The decision to administer diazepam was at the discretion of the treating physician and was not randomised or standardised. The sample size of 72 patients, while sufficient to detect the large effect size observed in the rate control group, limits the precision of effect estimates. The wide confidence and credible intervals reflect this imprecision.

These findings generate a hypothesis that warrants testing in a prospective, randomised, controlled trial. Such a trial should specifically target patients receiving rate control therapy only and should incorporate continuous rhythm monitoring, systematic collection of AF duration and type, assessment of comorbidities and chronic medications, and follow-up for thromboembolic events.

## 5. Conclusions

Adjunctive diazepam was associated with a significantly higher rate of spontaneous rhythm conversion in AF patients receiving rate control therapy only, consistent with sympatholysis removing a key factor sustaining the arrhythmia. This effect was not observed when Class IC/III antiarrhythmics were co-administered, indicating that diazepam’s benefit is context-dependent and likely reflects relief from sympathetically driven AF perpetuation when a direct antiarrhythmic mechanism is absent.

These hypothesis-generating findings warrant prospective validation in randomised controlled trials, with particular attention to thromboembolic risk in patients who convert unexpectedly.

## Figures and Tables

**Table 1:** Baseline characteristics of all patients (*N* = 72).

Variable	*n*	Median	IQR	Range
Age (years)	69	69	61–75	34–92
Weight (kg)	72	85	75–90	60–110
Height (cm) *	71	175	163–180	155–190
BMI (kg/m^2^) *	71	27.3	25.9–29.3	19.6–35.2
Heart rate (bpm)	72	126	118–140	88–180
Systolic BP (mmHg)	72	140	120–150	80–190
Diastolic BP (mmHg)	72	80	70–90	40–130
Diazepam dose (mg) ^†^	32	5	5–5	2–10
Heart rate after treatment (bpm)	72	88	72–97	50–160
Systolic BP after treatment (mmHg)	71	130	120–140	95–170
Diastolic BP after treatment (mmHg)	71	80	70–85	55–105
Time to therapeutic goal (h) ^‡^	69	3.0	2.5–6.0	1.5–12.0

* One patient excluded due to data entry error (height recorded as 15 cm). ^†^ Among 32 patients who received diazepam. ^‡^ Three patients hospitalized before reaching the therapeutic goal were censored. IQR = interquartile range; BP = blood pressure; BMI = body mass index.

**Table 2:** Effect of diazepam stratified by treatment strategy: baseline characteristics and outcomes.

**A. Rhythm Control Group (** ***n*** **= 33)**			
Patients Receiving Any Class IC or III Antiarrhythmic (Flecainide, Amiodarone, or Propafenone)			
**Baseline Characteristics**			
**Variable**	**Diazepam**	**No Diazepam**	***p*** **-Value**
*n*	16	17	
Age (years), median (IQR)	65 (61–73)	63 (57–69)	0.348
Male sex, *n* (%)	5 (31.2%)	12 (70.6%)	0.038 *
Baseline HR (bpm), median (IQR)	128 (120–141)	124 (116–130)	0.187
Baseline SBP (mmHg), median (IQR)	132 (120–142)	130 (115–144)	0.744
**Outcomes**			
**Outcome**	**Diazepam**	**No Diazepam**	**OR (95% CI)**
Rhythm conversion, *n*/*N* (%)	9/14 (64.3%)	11/17 (64.7%)	0.98 (0.22–4.30)
HR < 100 bpm achieved, n (%)	12/16 (75.0%)	14/17 (82.4%)	0.64 (0.12–3.46)
HR < 110 bpm achieved, n (%)	13/16 (81.2%)	16/17 (94.1%)	0.27 (0.03–2.92)
**B. Rate Control Only Group (n = 39)**			
Patients receiving beta-blockers and/or digoxin only (no Class IC/III antiarrhythmics)			
**Baseline characteristics**			
**Variable**	**Diazepam**	**No Diazepam**	***p-Value***
*n*	16	23	
Age (years), median (IQR)	70 (63–74)	77 (66–84)	0.127
Male sex, *n* (%)	9 (56.2%)	10 (43.5%)	0.523
Baseline HR (bpm), median (IQR)	127 (114–140)	126 (116–144)	0.596
Baseline SBP (mmHg), median (IQR)	140 (125–152)	140 (129–150)	0.636
**Outcomes**			
**Outcome**	**Diazepam**	**No Diazepam**	**OR (95% CI)**
**Spontaneous conversion, ** ***n*** **/** ***N*** **(%)**	6/15 (40.0%)	2/21 (9.5%)	6.33 (1.06–37.78) *
HR <100 bpm achieved, *n* (%)	14/16 (87.5%)	15/23 (65.2%)	3.73 (0.67–20.69)
HR <110 bpm achieved, *n* (%)	15/16 (93.8%)	18/23 (78.3%)	4.17 (0.44–39.68)

* *p* < 0.05. Baseline comparisons: Mann–Whitney U test for continuous variables; Fisher’s exact test for categorical variables. Outcome comparisons: Fisher’s exact test with odds ratios (log-transform method); Mann–Whitney U for continuous variables. IQR = interquartile range; HR = heart rate; SBP = systolic blood pressure; OR = odds ratio; CI = confidence interval. Three patients did not reach therapeutic goal (1 I rate and 2 in rhythm control groups).

## Data Availability

The data supporting the reported results are available from the corresponding author upon reasonable request.

## Supplementary Materials

The following supporting information can be downloaded at: https://www.mdpi.com/article/10.3390/medicina62050861/s1, Table S1: Antiarrhythmic drugs administered; Table S2: Bayesian logistic regression results for rhythm conversion; Table S3: Bayesian logistic regression results for HR ≤ 110 bpm in rate control stratum; Figure S1: Marginal probability plots–rhythm conversion model; Figure S2: Calibration plot–rhythm conversion model; Figure S3: Marginal probability plots–heart rate model.

## Abbreviations

AF	Atrial fibrillation
AV	Atrioventricular
bpm	Beats per minute
CI	Confidence interval
CrI	Credible interval
DBP	Diastolic blood pressure
ECV	Electrical cardioversion
GABA	Gamma-aminobutyric acid
HR	Heart rate
IQR	Interquartile range
LVEF	Left ventricular ejection fraction
OR	Odds ratio
SBP	Systolic blood pressure
