# Supplementary material for: Effects of Diazepam Addition to Standard Treatment of Atrial Fibrillation in Emergency Department Settings: A Unicentric Retrospective Study
Source: Medicina (Kaunas). 2026 Apr 30;62(5):861. doi: 10.3390/medicina62050861 (PMC13208983; doi:10.3390/medicina62050861)
Supplement: Supplementary file 1 [file medicina-62-00861-s001.zip › FigureS3.pptx]

## Slide 1
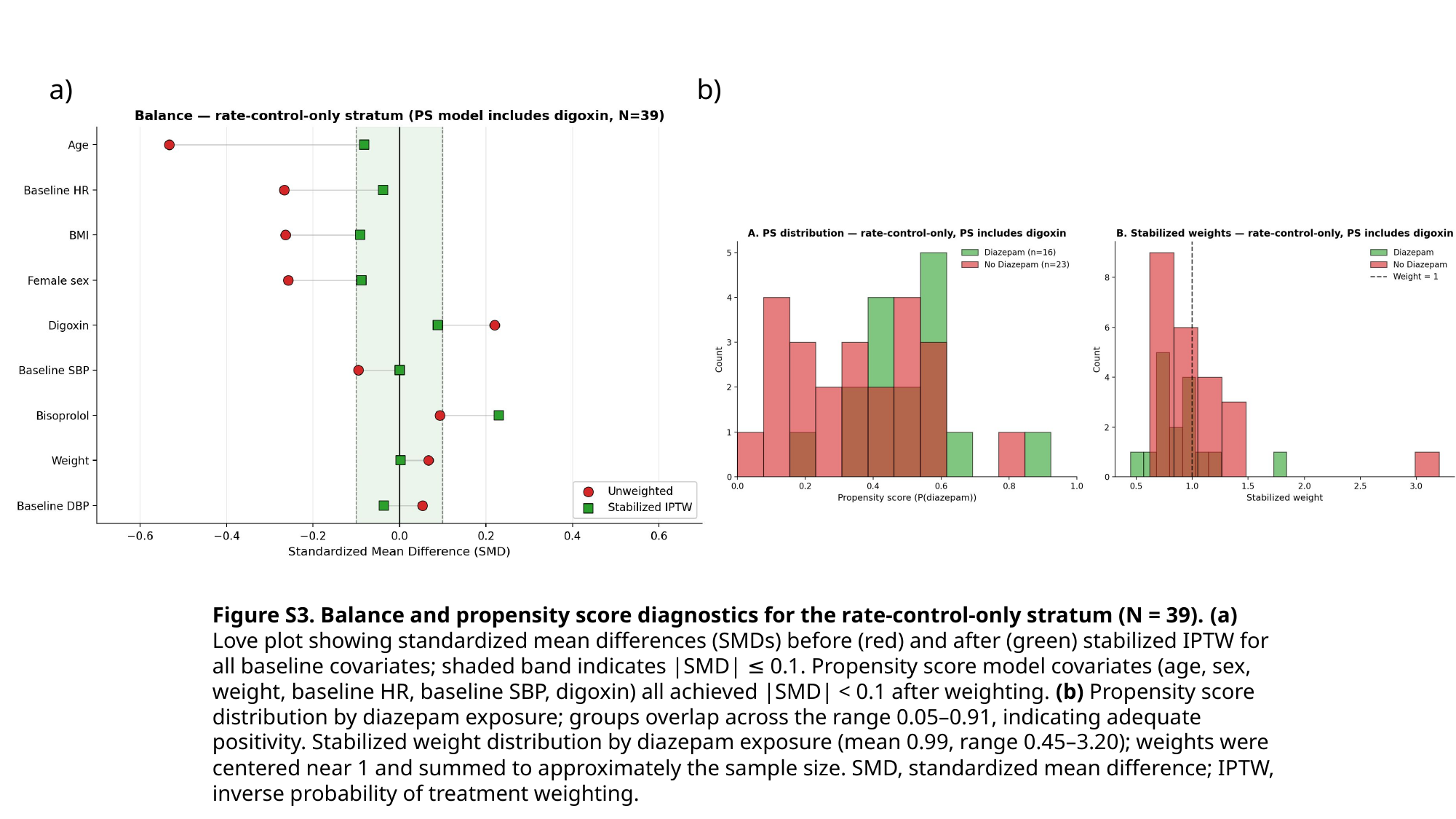

a)
b)
Figure S3. Balance and propensity score diagnostics for the rate-control-only stratum (N = 39). (a) Love plot showing standardized mean differences (SMDs) before (red) and after (green) stabilized IPTW for all baseline covariates; shaded band indicates |SMD| ≤ 0.1. Propensity score model covariates (age, sex, weight, baseline HR, baseline SBP, digoxin) all achieved |SMD| < 0.1 after weighting. (b) Propensity score distribution by diazepam exposure; groups overlap across the range 0.05–0.91, indicating adequate positivity. Stabilized weight distribution by diazepam exposure (mean 0.99, range 0.45–3.20); weights were centered near 1 and summed to approximately the sample size. SMD, standardized mean difference; IPTW, inverse probability of treatment weighting.
