# Supplementary material for: Effects of Diazepam Addition to Standard Treatment of Atrial Fibrillation in Emergency Department Settings: A Unicentric Retrospective Study
Source: Medicina (Kaunas). 2026 Apr 30;62(5):861. doi: 10.3390/medicina62050861 (PMC13208983; doi:10.3390/medicina62050861)
Supplement: Supplementary file 1 [file medicina-62-00861-s001.zip › Prior selection supplement.pdf]

Table S4. Priors for modelling rhythm conversion probability (log odds scale)

Variable	**Prior**	Implied prior belief
**Intercept (β_0)**	N(−2, 1)	Literature: ~10% spontaneous conversion
**Diazepam (β_1)**	N(0, 100)	Flat
**Class I/III (β_2)**	N(2.5, 1)	Literature: ~75% conversion with antiarrhythmics
**Interaction (β_3)**	N(0, 100)	Flat
**Age (β_4)**	N(0, 0.5)	Weakly informative
**Female (β_5)**	N(0, 0.5)	Weakly informative

Literarature:

Martinez-Marcos et al. (2000) — Am J Cardiol

Donovan et al. (1995) — Am J Cardiol

Table S5. Priors for modeling probability of HR<110 bpm (log odds scale)

**Variable**	**Prior**	**Implied prior belief**
**Intercept**	N (0, 2.5)	Weakly informative
**Diazepam**	N (0, 1.5)	Centered at null
**Age (centred)**	N (0, 2.5)	Weakly informative
**Sex (female)**	N (0, 2,5)	Weakly informative
