# Supplementary material for: Effects of Diazepam Addition to Standard Treatment of Atrial Fibrillation in Emergency Department Settings: A Unicentric Retrospective Study
Source: Medicina (Kaunas). 2026 Apr 30;62(5):861. doi: 10.3390/medicina62050861 (PMC13208983; doi:10.3390/medicina62050861)
Supplement: Supplementary file 1 [file medicina-62-00861-s001.zip › Suplementary tables.pdf]

Table S1. Antiarrhythmic drugs and their doses (A) (n = 72), the number of antiarrhythmics administered per patient (B) and stratification by treatment strategy (C)

A

**Drug**	**Class**	**n (%)**	**Doses used (mg)**
Bisoprolol	Beta-blocker	61 (84.7%)	2.5, 5, 7.5, 10
Digoxin	Cardiac glycoside	20 (27.8%)	0.2, 0.25, 0.3, 0.5, 0.75
Flecainide	Class IC	16 (22.2%)	150, 200, 300
Amiodarone	Class III	13 (18.1%)	300, 450
Propafenone	Class IC	4 (5.6%)	150, 300, 450
Carvedilol	Beta-blocker	1 (1.4%)	25

B

**Number of drugs**	**n (%)**
1 drug	31 (43.1%)
2 drugs	39 (54.2%)
3 drugs	2 (2.8%)

C

**Strategy**	**n (%)**
Rate control only	39 (54.2%)
Rhythm control only	33 (45.8%)

Rate control: beta-blockers (bisoprolol, carvedilol), cardiac glycosides (digoxin).
Rhythm control: Class IC (flecainide, propafenone), Class III (amiodarone).

Table S2. Bayesian logistic regression: predictors of rhythm conversion (n = 64 evaluable)

**Predictor**	**OR**	**95% CrI**	***P*(benefit, OR>1)**
Class IC/III antiarrhythmic	17.1	4.5–68.7	>99%
Diazepam (rate control reference)	3.86	0.61–23.3	92%
Diazepam × Class IC/III (interaction)	0.24	0.03–2.36	11%†
Age (per 10 years)	0.86	0.54-1.38	26%
Female sex	1.77	0.79-3.9	91%
Intercept	0.07	0.01-2.36	<1%

† Posterior probability of effect modification (interaction effect).

OR = odds ratio (posterior median); CrI = credible interval.

Model fit: Brier score: 0.172

Table S3. Bayesian logistic regression: predictors of achieving HR <110 bpm in the rate control stratum (n = 39)

Predictor	OR	95% CrI	*P*(benefit, OR>1)
Diazepam	3.46	0.63–23.1	92%
Age	2	0,77-5,65	92%
Female sex	0.34	0.03-3.42	18.2%

OR = odds ratio (posterior median); CrI = credible interval.
Model fit: Brier score= 0.128
