# Supplementary figures and images for: Effects of Diazepam Addition to Standard Treatment of Atrial Fibrillation in Emergency Department Settings: A Unicentric Retrospective Study
Source: Medicina (Kaunas). 2026 Apr 30;62(5):861. doi: 10.3390/medicina62050861 (PMC13208983; doi:10.3390/medicina62050861)

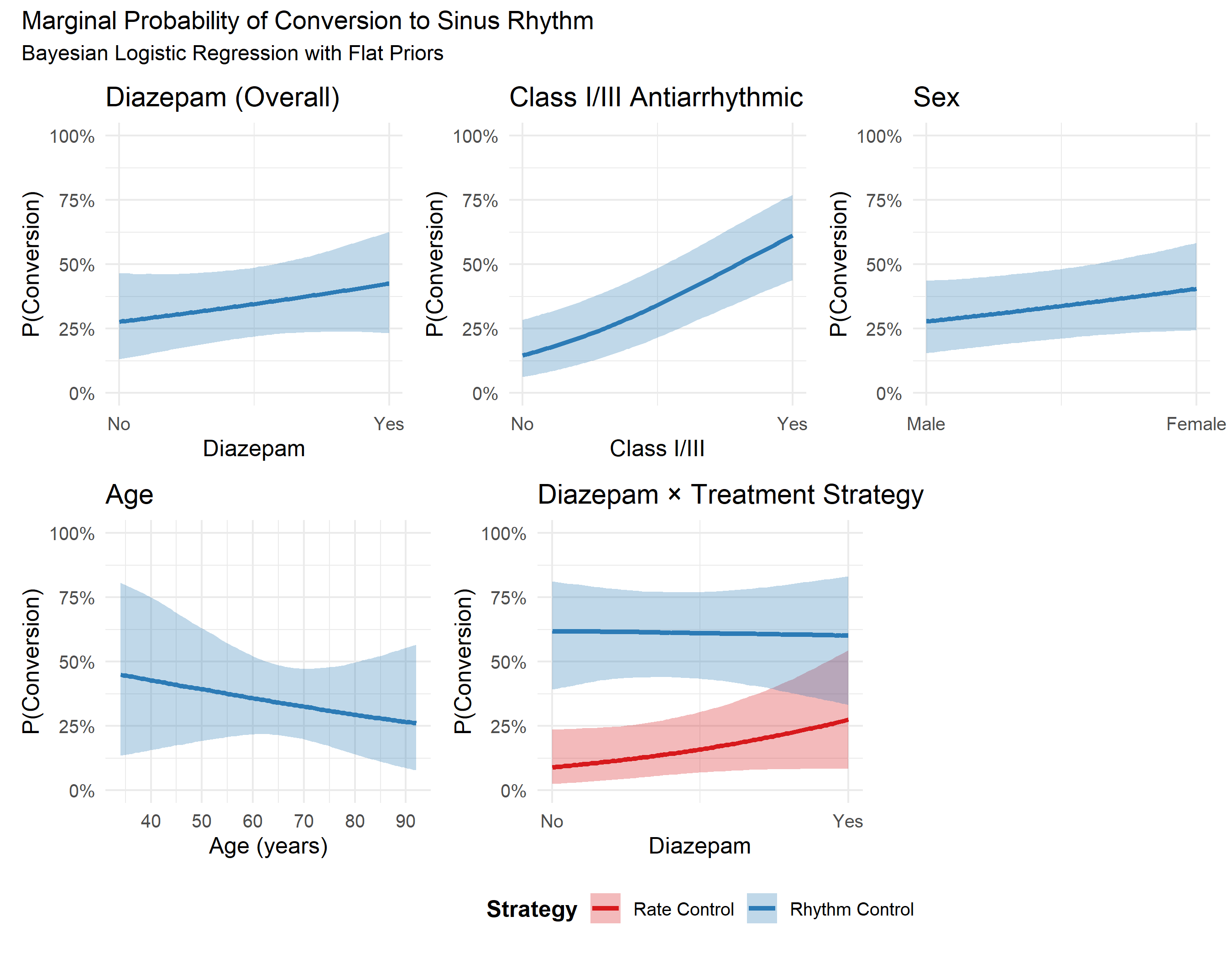

Supplement: Supplementary file 1 [file medicina-62-00861-s001.zip › FigureS1.png]

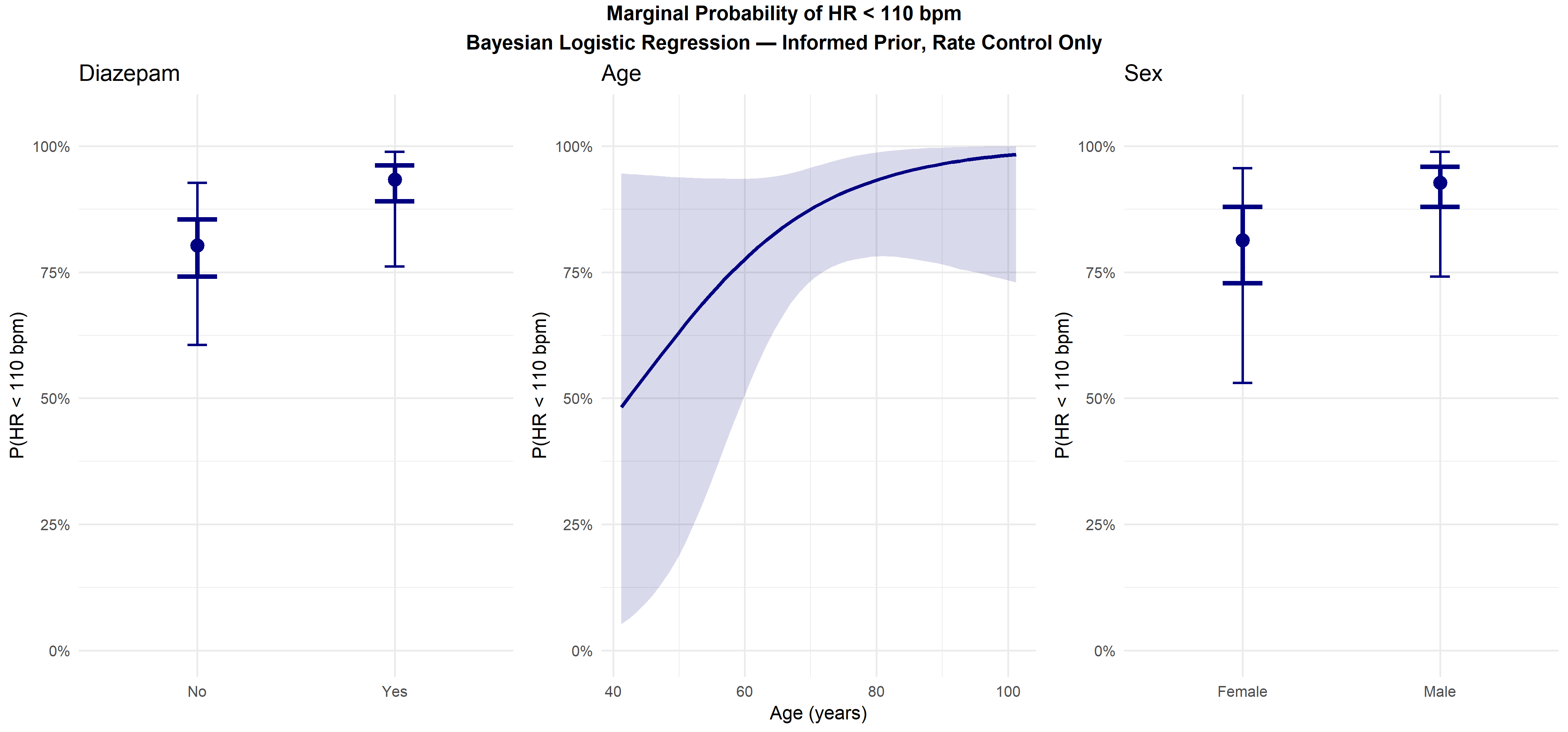

Supplement: Supplementary file 1 [file medicina-62-00861-s001.zip › FigureS2.png]
